# Proteolytic degradation of Beta-Ig H3 (βigH3/TGFBI) can be quantified non-invasively in serum and predicts prognosis in patients with advanced pancreatic ductal adenocarcinoma

**DOI:** 10.1186/s12885-025-14283-w

**Published:** 2025-05-20

**Authors:** Rasmus S. Pedersen, Annika Hettich, Jeppe Thorlacius-Ussing, Lasse L. Langholm, Marina Crespo-Bravo, Inna M. Chen, Carsten P. Hansen, Julia S. Johansen, Hadi M. H. Diab, Lars N. Jorgensen, Morten Karsdal, Nicholas Willumsen

**Affiliations:** 1 Nordic Bioscience A/S, 2730 Herlev, Denmark; 2 Department of Biomedical Sciences, University of Copenhagen, 2200 Copenhagen, Denmark; 3 Department of Oncology, Copenhagen University Hospital - Herlev and Gentofte, 2730 Herlev, Denmark; 4 Department of Surgery, Copenhagen University Hospital - Rigshospitalet, 2100 Copenhagen, Denmark; 5 Department of Medicine, Copenhagen University Hospital - Herlev and Gentofte, 2730 Herlev, Denmark; 6 Department of Clinical Medicine, Faculty of Health and Medical Sciences, University of Copenhagen, 2200 Copenhagen, Denmark; 7 Digestive Disease Center, Copenhagen University Hospital - Bispebjerg and Frederiksberg, 2400 Copenhagen, Denmark

## Abstract

The extracellular matrix (ECM) protein Beta-Ig H3 (βigH3, also known as transforming growth factor β induced protein (TGFBI)) is related to poor prognosis in patients with pancreatic ductal adenocarcinoma (PDAC). Proteolytic cleavage of βigH3 has been shown to result in release of the N-terminal fragment covering amino acid 1 to 137, but whether the degradation of βigH3 is associated to prognosis has yet to be determined. In this study we developed an ELISA targeting a collagenase generated fragment of βigH3 (cβigH3) in human serum to use the fragment as a biomarker reflecting degradation of βigH3. We demonstrated that the assay was specific to the cleaved fragment (cβigH3) and confirmed the generation of cβigH3 from degradation of fibroblast generated matrices. Moreover, higher levels of cβigH3 were released upon degradation of matrices produced by TGF-β stimulated pancreatic fibroblast compared to matrices produced by pancreatic fibroblast without TGF-β stimulation, indicating an association of the biomarker with degradation of fibrotic matrix. To evaluate the clinical relevance, we first measured cβigH3 in a cohort of 220 patients with different types of cancer with detectable levels for all 11 cancer types. We then measured the cβigH3 biomarker in pre-treatment serum from a cohort of 469 patients with locally advanced or metastatic PDAC and found that high levels of cβigH3 were associated with longer overall survival independently of age, disease stage, performance status, carbohydrate antigen 19–9 (CA19-9), and the tumor fibrosis biomarker PRO-C3 as compared to patients with high levels of cβigH3 (HR 0.78, 95% CI: 0.0.61–0.98, *p* = 0.04). In conclusion, cβigH3 reflects proteolytic degradation of βigH3 and shows potential as an independent prognostic biomarker for patients with advanced PDAC.

**Supplementary Information:**

The online version contains supplementary material available at 10.1186/s12885-025-14283-w.

## Introduction

The tumor microenvironment (TME) has been shown to play a major part in the development, progression, and treatment of cancer [1–4]. The TME is a dynamic niche with cross-talk between cellular and non-cellular components [1, 4–7]. The cellular components of the TME include cancer cells, stromal cells, cancer associated fibroblasts (CAFs), macrophages, and other immune cells [1, 4, 6]. The non-cellular components consist of the extracellular matrix (ECM), exosomes, and various proteins [1, 8, 9]. The ECM undergoes constant remodeling with formation, degradation, and modification of various proteins which, if balanced, are key to maintaining homeostasis in healthy tissues [10]. During cancer progression this balance can be skewed towards formation leading to accumulation of matrix proteins especially collagens. This concept is referred to as tumor fibrosis or desmoplasia and is thought to be driven by activation of fibroblasts that differentiate into CAFs, which is often stimulated by transforming growth factor beta (TGF-β), a cytokine commonly shown as an inducer of fibrosis across diseases [11–14]. Desmoplasia has been shown to be associated with poor treatment response and survival of patients across several cancer types [10, 15–18].

Pancreatic ductal adenocarcinoma (PDAC) is known as one of the most fibrotic cancers with the stroma accounting for the majority of the tumor volume [19]. With a 5-year overall survival rate of only 12%, PDAC is one of the leading causes of cancer deaths [20]. The extensive fibrosis of PDAC tumors is a major factor for the low survival rate by influencing tumor development, progression, metastasis, and treatment resistance as it acts as a barrier around the tumor limiting access for chemo- and immunotherapy [18, 21, 22]. The gold standard for assessing tumor fibrosis is by tissue biopsy followed by collagen staining, but serological quantification of specific collagen fragments is proving to be a promising and less invasive solution [22–24]. One of these fragments is the pro-fragment of type III collagen (PRO-C3), which reflects formation of type III collagen [15]. Previous studies have shown that high serum levels of PRO-C3 are associated with short survival in patients with PDAC [14, 24, 25].

The characteristics of the desmoplasia surrounding PDAC tumors are not exclusively related to the amount of collagen but also the structuring of these within the ECM [26–30]. Beta-Ig H3 (βigH3) (also known as TGF-β induced protein (TGFBI)) is a protein located in the matrix and has been shown to be involved in structuring the ECM through binding to various matrix proteins including type I and type III collagen [27, 31]. While some studies show evidence that βigH3 is tumor suppressive in early tumor development, several studies have seen βigH3 expression correlating to poor patient outcome with increased metastatic potential, decreased treatment response and decreased overall survival [32–36]. Various cell types have been shown to express βigH3 upon stimulation with TGF-β including fibroblasts, macrophages, and different types of cancer cells [37–40]. In addition to structuring collagens in the ECM, βigH3 acts as a linker between cell surfaces and ECM components and is involved in activation of several intracellular pathways including AKT, ERK, FAK, and Paxilin [41, 42]. While βigH3 is mainly localized in the matrix, several studies have shown potential for quantification of βigH3 in circulation as a biomarker in both non-cancerous and cancerous diseases. In these studies βigH3 was elevated in disease and often associated with poor outcome [33, 42–45]. With the tumor-promoting role of βigH3 it can be speculated that degradation of βigH3 could be beneficial for the patient and quantification thereof could provide additional value. Enzymatic processing of βigH3 has been confirmed and in addition different cleaved forms of βigH3 have been shown to have different functions, indicating that the specific cleavage sites might lead to certain properties [46, 47]. Kim et al*.* [47] identified a cleavage site of βigH3 between the amino acid S^137^ and P^138^ using mass spectrometry, which should result in the release of the N-terminal part of the protein hereafter referred to as cβigH3 (cleaved βigH3) into circulation. In addition to identifying the cleavage site they also showed indication that cleavage of βigH3 induced cell migration, while full length βigH3 did not, thus suggesting that cleavage of βigH3 is involved in biological processes. To further investigate the role of cleaved βigH3 and how it affects patient outcome more accessibly methods than mass spectrometry is needed.

In this study we developed an ELISA targeting cβigH3 in human serum to use the fragment as a biomarker reflecting degradation of βigH3. We first evaluated technical performance and specificity of the assay and measured the cβigH3 biomarker across various solid tumor types. We then investigated the diagnostic and prognostic potential of cβigH3 for patients with advanced PDAC and the association with the tumor fibrosis biomarker PRO-C3.

## Methods

### Target identification antibody production and neoepitope specificity

We produced antibodies against the amino-acid peptide ^128^LRPEMEGPGS^137^ corresponding to the C-terminal of a fragment of βigH3 (cβigH3), generated upon degradation, which was identified by Kim et al. [47] by immunizing six-week-old Balb/C mice with the immunogenic peptide; the target peptide sequence with a cysteine and glycine linker to keyhole limpet hemocyanin (KLH) carrier protein ((KLH)-CGG-LRPEMEGPGS) as described in Pedersen et al. [48].

We investigated potential non-specific biding by using BLAST on the amino-acid sequence with NPS@: Network Protein Sequence Analysis with UniportKB/Swiss-prot database. Based on homology to the target sequence, origin, location, and biological function, we identified two relevant potential off-targets for the antibody: EAPVTEGPGS (from ADAMTS13) and PCPCPEGPGS (from LAMB2).

To confirm epitope specificity of the antibody, we used it in a competitive ELISA with biotinylated selection peptide as coater (Biotin-K-LRPEMEGPGS), and two-fold dilution series of the 10 and 30 amino acid selection peptides (LRPEMEGPGS and ETLGVVGSTTTQLYTDRTEKLRPEMEGPGS), elongated peptide (LRPEMEGPGSF), truncated peptide (LRPEMEGPG) and the two peptides with the sequences from the identified potential off-targets (deselection peptides) (EAPVTEGPGS and PCPCPEGPGS). All peptides used in this study were purchased from Genscript (Piscataway, NJ, USA).

### Assay development and evaluation

We developed a competitive ELISA with the cβigH3-targeting antibodies hereafter referred to as nordicBIGH3M-N. We evaluated the technical performance of the nordicBIGH3M-N. The standards curve for the assay was made with a 4-point logistic curve fit of a twofold dilution of 30 amino acid selection peptide. The assay underwent several technical tests to evaluate the following aspects of the assay: Establishment of quantification range, dilutional linearity, spiking accuracy, inter- and intra- assay variation, tolerance to interference from hemoglobin, lipids, and biotin, and finally analyte and kit stability. To determine the lower limit of quantification (LLOQ) four human serum samples with cβigH3 levels close to the expected lower limit of measurement range and with CV% values around 20% were run in six replicates in five independent runs. The LLOQ was defined as the cβigH3 levels resulting in CV% of 20% based on a power regression model made from cβigH3 levels and CV% for the four samples (Supplementary Fig. 1).

To evaluate the inter- and intra- assay variation and establish upper limit of quantification (ULOQ) we used 10 samples consisting of seven human serum samples, one sample consisting of pooled human serum samples and two human serum samples spiked with 30 amino acid selection peptide. The 10 samples were run in duplicates in 10 independent runs. The ULOQ was determined as the concentration of the highest point of the standard curve with a relative error percent (RE%) of less than 20% in more than 80% of the samples. The inter- and intra-assay variation was estimated based on CV% of the 10 samples within the same plate and across the different plates, respectively. The inter-assay variation was the mean CV% of the 10 samples between the 10 runs, and the intra-assay variation was defined as the average CV% between sample duplicates on the same plate. Additionally, the IC50 of the assay were established as the average IC50 from the curve fit from these 10 runs.

Four human serum samples serially diluted twofold were measured in 3 independent runs and the RE% relative to all lower dilutions was calculated to establish the minimum required dilution (MRD) and evaluate linearity. MRD was determined as the lowest dilution where further twofold dilution had RE% between 80–120%. For spiking accuracy, we assessed the recovery from spiking three human serum samples with a twofold serial dilution of 30 amino acid peptide. In addition, we spiked three human serum samples with low levels of cβigH3 with three human serum samples with higher levels of cβigH3 and calculated the RE% between measured levels and expected levels based on measured levels in the pure samples and the spiking ratio. Three human serum samples were spiked with low and high levels of the known interfering substances hemoglobin (2.5 mg/mL and 5 mg/mL, respectively), lipids (1.5 mg/mL and 5 mg/mL, respectively), and biotin (5 ng/mL and 100 ng/mL, respectively) and recovery relative to control samples were calculated.

To assess the stability of the analyte a stress test and a freeze–thaw test were performed for three human serum samples for which recovery was calculated using the non-stressed sample as reference. For the stress test, the samples were measured after being stored at 4 °C, 20 °C, and 37 °C for 4, 24, and 48 h for each temperature, with fresh aliquots of the samples as reference. For the freeze–thaw test, the samples were thawed at 20 °C for one hour and frozen again for at least 24 h with 1–5 freeze–thaw cycles and fresh aliquots as reference. A stress test and freeze–thaw test of the ELISA kit reagents were also performed. For the stress test, all kit reagents were stored at 4 °C, 20 °C, and 37 °C for one, three, and seven days for each temperature with a fresh kit as reference. Five serum samples were measured in the stress test and recovery was calculated using the non-stressed kit as reference. For the freeze–thaw test, the main reagents of the assay (biotinylated-peptide, selection peptide, control samples, and antibody) was thawed at 20 °C for one hour and frozen again for at least 24 h with 1–3 freeze–thaw cycles and a fresh kit as reference. Five human serum samples were measured in each of the 4 kits and recovery was calculated using the non-stressed kit as reference.

### nordicBIGH3M-N ELISA protocol

During assay development multiple variables were changed and optimized to reach the final assay protocol. These variables included: assay buffer, incubation time and temperature, biotinylated peptide/antibody ratio and antibody conjugation of horseradish peroxidase (HRP). For the final nordicBIGH3M-N ELISA protocol 96-well streptavidin-coated plates were coated with 100 µl of 6 ng/mL biotinylated selection peptide (Biotin-K-LRPEMEGPGS) and incubated shaking with 300 revolutions per minute (rpm) in darkness at 20 °C for 30 min followed by a washing of the wells (5 washes with washing buffer (20 mM TRIS, 50 mM NaCl, pH 7.2)). After washing, 20 µL of a twofold serial dilution (in assay buffer) of 30 amino acid std peptide (ETLGVVGSTTTQLYTDRTEKLRPEMEGPGS) starting from 100 nM, 20 µL of serum samples (or in vitro cleavage samples), 20 µL of two control samples and three quality control samples were added to the plate. 100 µL of 10 ng/mL cβigH3-targeting HRP-labelled antibody was added to each well and the plates were incubated shaking with 300 rpm in darkness at 4 °C for 20 h (± 1 h). The plates were then washed 5 times with washing buffer and 100 µL of TMB (Kem-En-Tec Diagnostics (Cat. No. 4380)) was added to each well and incubated in darkness at 20 °C for 15 min, after which the reaction was stopped by addition of 100 µL of 1% sulfuric acid. Finally, the plates were analyzed using a VersaMax ELISA microplate reader (Molecular Devices, San Jose, CA, USA) at 450 nm, with 650 nm as reference. We then used SoftMax Pro (version 7.1) to generate a standard curve and analyzed the data using GraphPad Prism (version 10). Biotinylated peptide, antibody, and standard peptide were diluted in assay buffer (50 mM PBS-BTB 4 g/L NaCl, pH 7.4) to the concentration used.

### Assessment of PRO-C3

PRO-C3 was measured using nordicPRO-C3 (cat#1700 AF01) according to the manufacturer’s instructions (Nordic Bioscience A/S, Herlev, Denmark). The procedure was similar to the cβigH3 protocol with coating of 96-well streptavidin-coated plates were coated with 100 µl of biotinylated selection peptide and incubated for 30 min shaking with 300 revolutions per minute (rpm) in darkness at 20 °C followed by a washing step as described for the cβigH3 protocol. After washing, 20 µL of a twofold serial dilution (in assay buffer) of std peptide, 20 µL of serum samples, 20 µL of two control samples, and three quality control samples were added to the plate as well as 100 µL of HRP-labelled antibody in assay buffer and the plate was incubated for 20 h (± 1 h) at 4 °C shaking with 300 rpm in darkness.

100 µL of 10 ng/mL cβigH3-targeting HRP-labelled antibody was added to each well and The plates were then washed 5 times with washing buffer and 100 µL of TMB (Kem-En-Tec Diagnostics (Cat. No. 4380)) was added to each well and incubated in darkness at 20 °C for 15 min, after which the reaction was stopped by addition of 100 µL of 1% sulfuric acid. Lastly, the plates were analyzed using a VersaMax ELISA microplate reader (Molecular Devices, San Jose, CA, USA) at 450 nm, with 650 nm as reference.

### Fibroblast cell culture and cleavage of matrices

The fibroblast cell culture protocol employed in this study was adapted from the method outlined by Chen et al. [49]. and Nissen et al. [14]. Native human pancreatic quiescent fibroblasts (cat# SC00 A5, Vitro Biopharma, Golden, CO, USA) were expanded in culture flasks coated with 5 µg/cm^2^ type I collagen purified from rat tail tendon (cat# P8188, Innoprot, Derio, Biscay, Spain). Upon reaching 80–90% confluency, cells were harvested and seeded in 48-well plates at a density of 30,000 cells per well. Subsequent cultivation was performed in Gibco DMEM + GlutaMAX (cat# 31,966,047, Thermo Fisher Scientific, Waltham, MA, USA), supplemented with 10% fetal bovine serum (FBS) (cat# F7524, Sigma-Aldrich, St. Louis, MO, USA) and 1% penicillin/streptavidin (P/S) (cat#P4333, Sigma-Aldrich, St. Louis, MO, USA). After 24 h, the medium was replaced with ficoll media, comprising 50% DMEM + GlutaMAX supplemented with 0.4% FBS, 1% P/S, and 50% 70 and 400 kDa Ficoll (cat#17,031,050 and cat#17,030,050, Cytvia, Marlborough, MA, USA) dissolved in DMEM + GlutaMAX supplemented with 0.4% FBS, 1% P/S, and 0.05 mg/mL of L-ascorbic acid (cat#013–12061, Fujifilm, Tokyo, Japan). Cells were either untreated or treated with 1 ng/ml TGF-β1 (cat# 7754-BH/CF, Bio-techne, Minneapolis, MN, USA). Media were exchanged every 3 days. On day 12, the extracellular matrices were decellularized. Wells were washed with PBS w/o CaCl_2_ and MgSO_4_ (PBS-) (cat# D8537, Sigma-Aldrich, St. Louis, MO, USA). Subsequently, 450 µl of extraction buffer (PBS- + 0.5% Triton X-100 + 20 mM NH_2_OH) was gently added to the wells without touching the cells and incubated for 10–15 min at 37 °C. After this, 450 µl of PBS- was added, and wells were stored overnight at 4 °C. The next day, cell debris was carefully removed, and wells were washed once with PBS- and then once with PBS containing 1 mM CaCl2 and 1 mM MgSO4 (PBS +).

To facilitate collagenase digestion of the matrices, all wells were washed with digestion buffer (0.05 M Tris, 0.36 mM CaCl_2_, pH 7.5) to calibrate the matrices. Subsequently, 200 µl of digestion buffer containing 0.1 mg/ml collagenase (cat# C9891, Sigma-Aldrich, St. Louis, MO, USA) was added to the wells and incubated for 72 h at 37 °C. Half of the wells were incubated in digestion buffer without collagenase to serve as controls. The enzymatic reaction was stopped with cOmplete Mini Protease Inhibitor Cocktail (cat# 11,836,153,001, Basal, Switzerland) for 5 min at room temperature. Digested matrices were stored at −20 °C until subsequent analysis.

### Subjects

We measured the cβigH3 biomarker using the nordicBIGH3M-N ELISA in serum samples from two cohorts of patients with cancer and one cohort of healthy individuals. Serum samples from healthy individuals were obtained from the commercial vendor BioIVT (Westbury, NY, USA) with an appropriate institutional review board/independent ethical committee-approved sample collection. Cohort 1 consists of serum samples from 220 patients with the following cancer types (20 patients for each cancer type): bladder cancer, breast cancer, colorectal cancer, gastric cancer, head and neck cancer, lung cancer, melanoma, ovarian cancer, pancreatic cancer, prostate cancer, and renal cancer, and 28 age- and sex matched healthy individuals. The serum sample from patients with cancer in cohort 1 was obtained from Proteogenex (Los Angeles, CA, USA). Patient demographics for cohort 1 are shown in Table 1 and include: Diagnosis, sex, age, and stage (American Joint Commission on cancer 8 th edition).

**Table 1:** Patient demographics and clinical profile for patients with 11 different types of solid cancer and healthy individuals (cohort 1)

Clinical variables	Patients with cancer (*n* = 220)	Healthy individuals (*n* = 28)
**Diagnosis**		
Healthy		28 (100%)
Bladder cancer	20 (9%)	
Breast cancer	20 (9%)	
Colorectal cancer	20 (9%)	
Gastric cancer	20 (9%)	
Head and neck cancer	20 (9%)	
Lung cancer	20 (9%)	
Melanoma	20 (9%)	
Ovarian cancer	20 (9%)	
Pancreatic cancer	20 (9%)	
Prostate cancer	20 (9%)	
Renal cancer	20 (9%)	
**Sex**, n		
Male	119 (54%)	20 (71%)
Female	101 (46%)	8 (29%)
**Age** (years), median (min–max)	57 (49–69)	57 (50–69)
**Stage**		
1	7 (3%)	
2	46 (21%)	
3	93 (42%)	
4	74 (34%)	

Cohort 2 consists of 469 patients with locally advanced (stage 3) or metastatic (stage 4) PDAC and 28 healthy individuals. Patient demographics for cohort 2 are shown in Table 2 and include: Sex, age, BMI, disease stage (American Joint Commission on cancer 8 th edition), diabetes, tobacco, and alcohol usage (a high usage corresponds to above Danish health authorities’ recommendations (DHAR)), carbohydrate antigen 19–9 (CA19-9) levels, and performance status (PS). The patients in cohort 2 had histologically confirmed PDAC and received treatment according to the Danish national guidelines. Serum samples were obtained before the first treatment with palliative chemotherapy.

**Table 2:** Patient demographics and clinical profile for patients with locally advanced or metastatic PDAC and healthy individuals (cohort 2)

Clinical variables	Patients with PDAC (*n* = 469)	Healthy individuals (*n* = 28)
**Age,** years, Median (min–max)	68 (38–88)	48 (29–67)
**Sex**, n		
Male	250 (53%)	15 (54%)
Female	219 (47%)	13 (46%)
**BMI**, kg/m^2^, median (min–max)	23.2 (13.7–39.0)	Na
**Stage, n**		
3	192 (41%)	Na
4	277 (59%)	
**Diabetes, n**		
Yes	128 (27%)	Na
No	341 (73%)	
**Tobacco, n**		
Ever	155 (33%)	Na
Never	286 (61%)	
Unknown	28 (6%)	
**Alcohol, n**		
< DHAR	349 (74%)	Na
> DHAR	91 (19%)	
Unknown	29 (6%)	
**CA19-9 (U/mL), n**		
≤ median (≤ 704 U/mL)	234 (50%)	Na
> median (> 704 U/mL)	233 (50%)	
Unknown	2 (0%)	
**Performance status, n**		
0	145 (31%)	Na
1	213 (45%)	
2	48 (10%)	
3	3 (1%)	
Unknown	60 (13%)	

*Abbreviations: BMI* body mass index, *CA19-9* carbohydrate antigen 19–9, *DHAR* Danish Health Authority recommendations on alcohol consumption, *PDAC* pancreatic ductal adenocarcinoma, *Na* not available

The serum samples from patients used in cohort 2 were collected between July 2008 and June 2020, and the patients were followed until August 2023 or death, whichever came first at the time of clinical data collection. All samples in both cohorts were measured blinded to clinical information. Serum samples were stored at −80 °C from collection to biomarker measurement.

### Statistics

For cohort 1 cβigH3 levels of each cancer type were compared to those of healthy subjects using the Kruskal–Wallis test followed by Dunn’s test of multiple comparisons test. For cohort 2 cβigH3 levels for patients with PDAC were compared with healthy subjects using unpaired T-test. cβigH3 levels for patients divided into low/high PRO-C3 levels were compared using Mann–Whitney test. Survival analysis and calculation of hazard ratio (HR) were performed using log-rank test and Cox proportional hazard regression. Significance was considered with p-values < 0.05 and depicted as follows: *ns* *p* > 0.05, * *p* < 0.05, ** *p* < 0.01, *** *p* < 0.001, and **** *p* < 0.0001. Statistical analysis and graphic illustrations were performed using GraphPad Prism (version 10), GraphPad Software, San Diego, California, USA and MedCalc (version 19.3), MedCalc Software Ltd, Ostend, Belgium.

## Results

### Technical evaluation of the cβigH3 ELISA assay

The cβigH3 ELISA was evaluated through various technical tests to determine specifications and the quality of the assay. The results of the technical evaluation are summarized in Table 3. We found a lower limit of detection of 0.79 nM and an IC50 of 5.76 nM with the quantification range for serum (LLOQ-ULOQ) of 1.06–100 nM. We were able to dilute human serum samples with an average dilution recovery of 118% from undiluted to 1:2 dilution and 89% from 1:2 to 1:4 dilution, indicating that the assay has dilution linearity but does not require dilution to be measurable. We investigated spiking accuracy and saw an average spiking recovery 95% for serum in serum spiking and 97% for 30 amino acid selection peptide in serum spiking. Inter- and intra-assay variation for serum sample measurements was 9.0% and 5.6%, respectively. We then examined the influence of high levels of three known serological interference compounds namely hemoglobin, biotin, and lipids, by adding them to the serum samples. We saw recoveries of 101%/103%, 101%/111%, and 102%/96% for low/high concentrations of hemoglobin, biotin, and lipids, respectively. Furthermore, we analyzed the analyte stability and saw that it was stable for at least 48 h at 4 °C, 20 °C, and 37 °C with a recovery of 111%, 98%, and 91% respectively. In addition, we found that the analyte was stable after five freeze/thaw cycles with a recovery of 90%. Finally, we investigated the stability of the ELISA kit and found that it was stable for 7 days at 4 °C and 20 °C and for 1 day at 37 °C, with recoveries of 98%, 92%, and 84%, respectively, and that key kit reagents were stable for up to three freeze thaw cycles, with a recovery of 113%. Together these results demonstrate that the cβigH3 ELISA assay is technically robust, stable, and appropriate for measuring cβigH3 in human serum samples.

**Table 3:** Summary of technical evaluation of cβigH3 ELISA assay

Test	Results
Quantification range (LLOQ-ULOQ)	1.06–100 nM
Lower limit of detection	0.79 nM
IC50	5.76 nM
Dilution recovery (undiluted➜1:2, 1:2➜1:4)	118%, 89%
Spiking recovery (serum in serum, peptide in serum)	95%, 97%
Inter-assay variation	9.0%
Intra-assay variation	5.6%
Interference (hemoglobin, low/high conc.)	101%/103%
Interference (biotin, low/high conc.)	101%/111%
Interference (lipids, low/high conc.)	102%/96%
Analyte stability (48 h 4 °C, 20 °C, and 37 °C)	111%, 98%, 91%
Analyte freeze/thaw stability up to five cycles	90%
Kit stability (7 days 4 °C, 20 °C, and 1 day 37 °C)	98%, 92%, 84%
Kit freeze/thaw up to 3 cycles	113%

### Confirmation of assay specificity towards cleaved BigH3

**Fig. 1:**
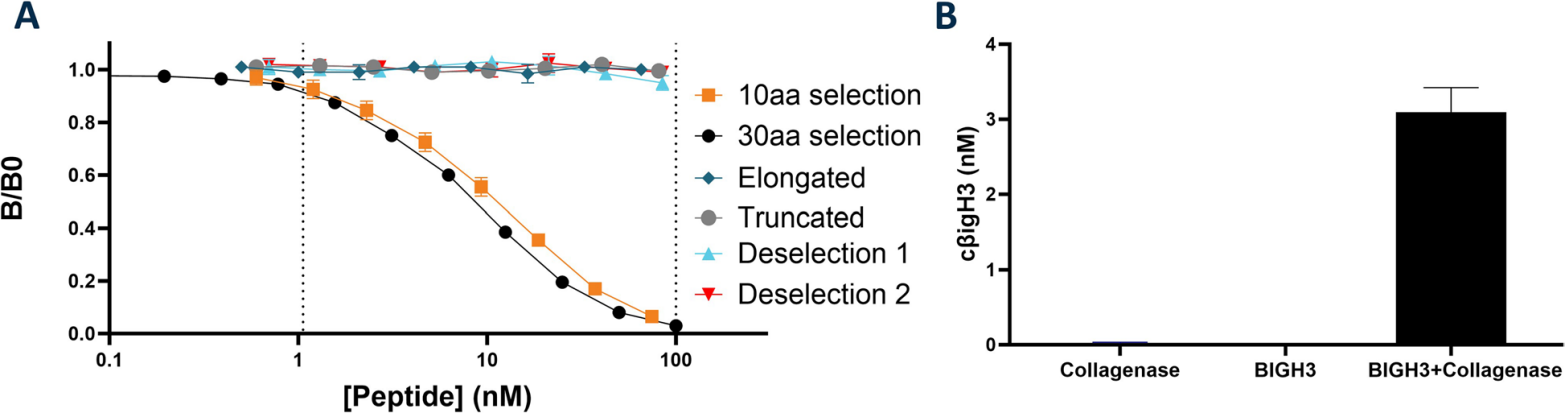
Assay specificity** (A**) Binding specificity towards neoepitope depicted as ratios of OD-values and MaxOD (B/B0) of twofold dilutions of 10 amino acid selection peptide (LRPEMEGPGS), 30 amino acid selection peptide (ETLGVVGSTTTQLYTDRTEKLRPEMEGPGS), elongated peptide LRPEMEGPGSF, truncated peptide (LRPEMEGPG), deselection peptide 1 (EAPVTEGPGS) and deselection peptide 2 (PCPCPEGPGS). **B** BIGH3M-N quantification after 24 h incubation at 37 °C of collagenase, BIGH3 recombinant protein, and the combination of the two (*n* = 2 and *N* = 1)

To confirm that we measured the specific cleaved fragment of βigH3 (cβigH3) we investigated whether the antibody could only detect the fragment resulting from cleavage of βigH3. To evaluate neoepitope specificity, we measured twofold dilutions of 10 and 30 amino acid selection peptide (^128^LRPEMEGPGS^137^ and ETLGVVGSTTTQLYTDRTEKLRPEMEGPGS), elongated peptide (^128^LRPEMEGPGSF^138^), truncated peptide (^128^LRPEMEGPG^127^), and two deselection peptides chosen from the sequence similarity to the target sequence and biological relevance (EAPVTEGPGS and PCPCPEGPGS) (Fig. 1A). No signal inhibition was observed for other than the selection peptides, indicating that the assay has neoepitope specificity towards the C-terminus of the N-terminal fragment upon cleavage of βigH3 at LRPEMEGPGS^137^↓.

To﻿ confirm that the cβigH3 fragment was indeed generated through proteolytic cleavage of βigH3, we incubated recombinant βigH3 with collagenase and measured it in the cβigH3 assay (Fig. 1B). The detection of cβigH3 was possible only when βigH3 and collagenase were incubated together, not when either of the two was incubated alone. This finding indicates that the fragment is produced through collagenase digestion of βigH3, providing confirmation that the cβigH3 assay measures a cleaved fragment of βigH3.

### βigH3 embedded in the matrix of pancreatic fibroblasts can be processed and quantified as cβigH3

**Fig. 2:**
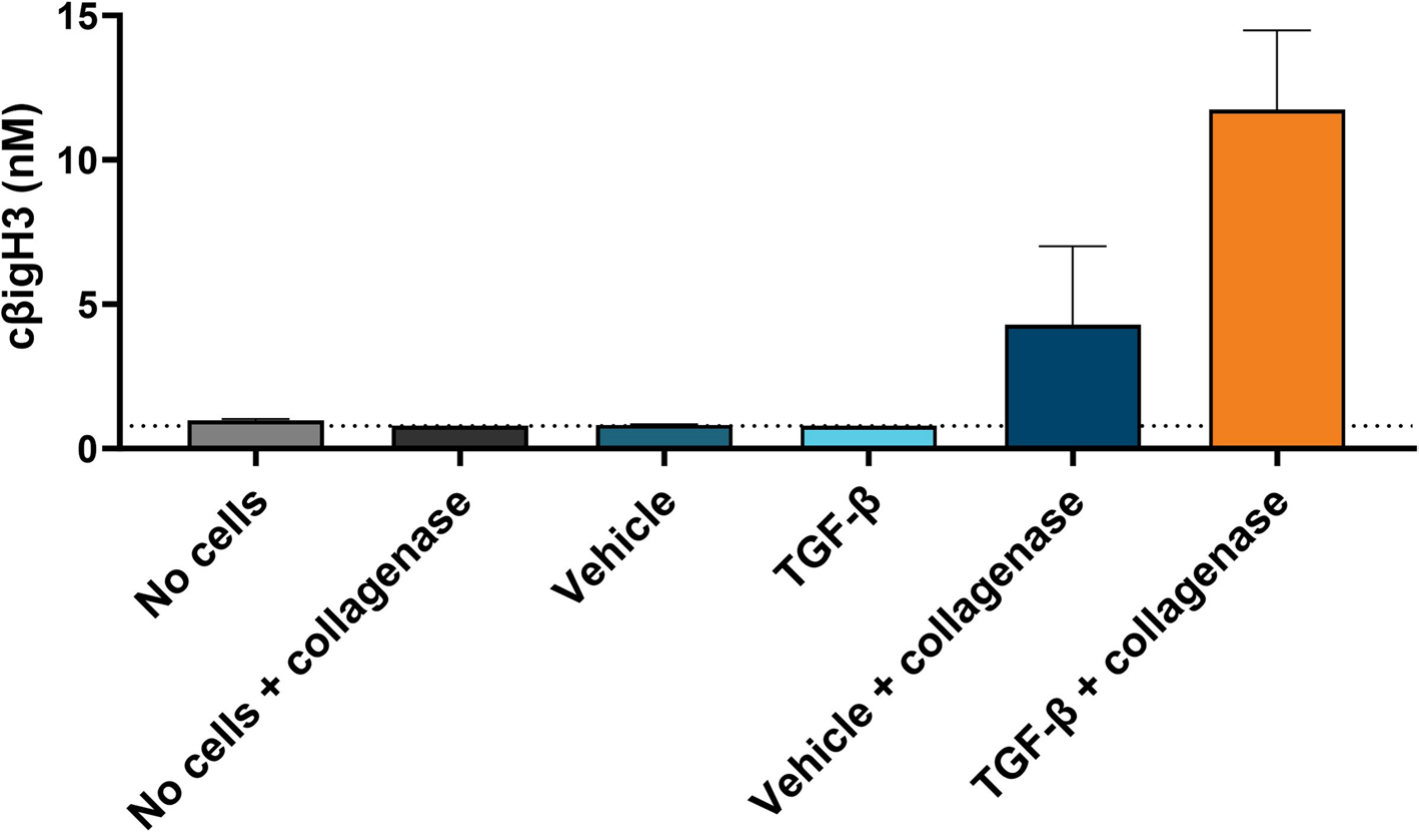
Matrix cleavage. cβigH3 levels measured in supernatant from pancreatic fibroblast generated matrixes after incubation with or without collagenase. Prior to collagenase cleavage the cells were treated with TGF-β or vehicle every three days for 12 days followed by decellularization, leaving the matrix generated from the cells. The data is from two independent experiments with two replicas in the first and six replicas in the second and is plotted as a mean value for each independent experiment. Values below LOB were set to LOB, which is indicated by the dotted line

To evaluate if the cβigH3 was associated with remodeling of ECM, we seeded pancreatic fibroblasts and kept them in culture without or with TGF-b treatment to generate fibrotic matrices. We decellularized the wells and degraded the matrices using collagenase and measured cβigH3. After collagenase degradation we could detect cβigH3, while nearly no cβigH3 could be detected for the controls without collagenase digestion (Fig. 2). Thus, demonstrating that βigH3 is produced by pancreatic fibroblasts and bound to matrix and that degradation of the matrices leads to release of the cβigH3 fragment, as well as further supporting the specificity of the assay towards processed βigH3. We saw a clear trend towards higher concentrations of cβigH3 for degraded matrices generated with TGF-β treatment (mean = 11.7 nM) compared to matrices generated without TGF-β treatment (mean = 4.3 nM) (Fig. 2), indicating that cβigH3 is associated with degradation of fibrotic matrices.

### cβigH3 could be detected in serum from patients with different types of cancer

**Fig. 3:**
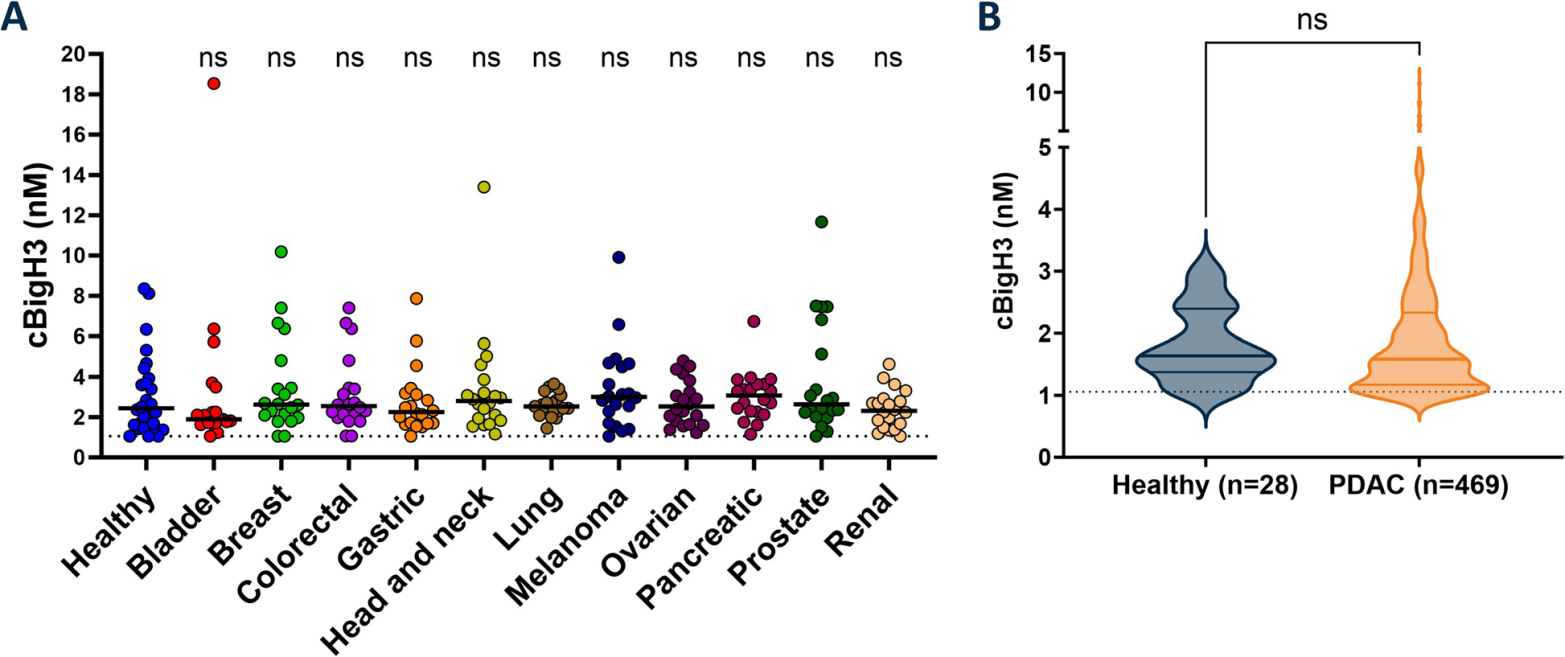
Diagnostic biomarker potential. **A** cβigH3 quantification in serum from patients in cohort 1 consisting of patients with different cancer types and healthy controls. All cancer types were compared to healthy controls using Dunn’s multiple comparisons test. **B** cβigH3 quantification in serum from patients in cohort 2 consisting of patients with advanced PDAC and healthy controls. The two groups were compared using unpaired t-test. Black line indicates median cβigH3 value of the sample group and the dotted line indicates lower limit of quantification. ns indicates *p* > 0.05

We measured cβigH3 in serum from cohort 1. We measured the fragment in almost all patients across cancer types but saw no significant difference in cβigH3 concentration between serum from the healthy individuals and the patients with either cancer type (Fig. 3A). Similarly, there was no significant difference in serum levels of cβigH3 between the healthy group and the patients with advanced PDAC (cohort 2) (Fig. 3B).

### cβigH3 were independently associated with survival outcome for patients with advanced PDAC

**Fig. 4:**
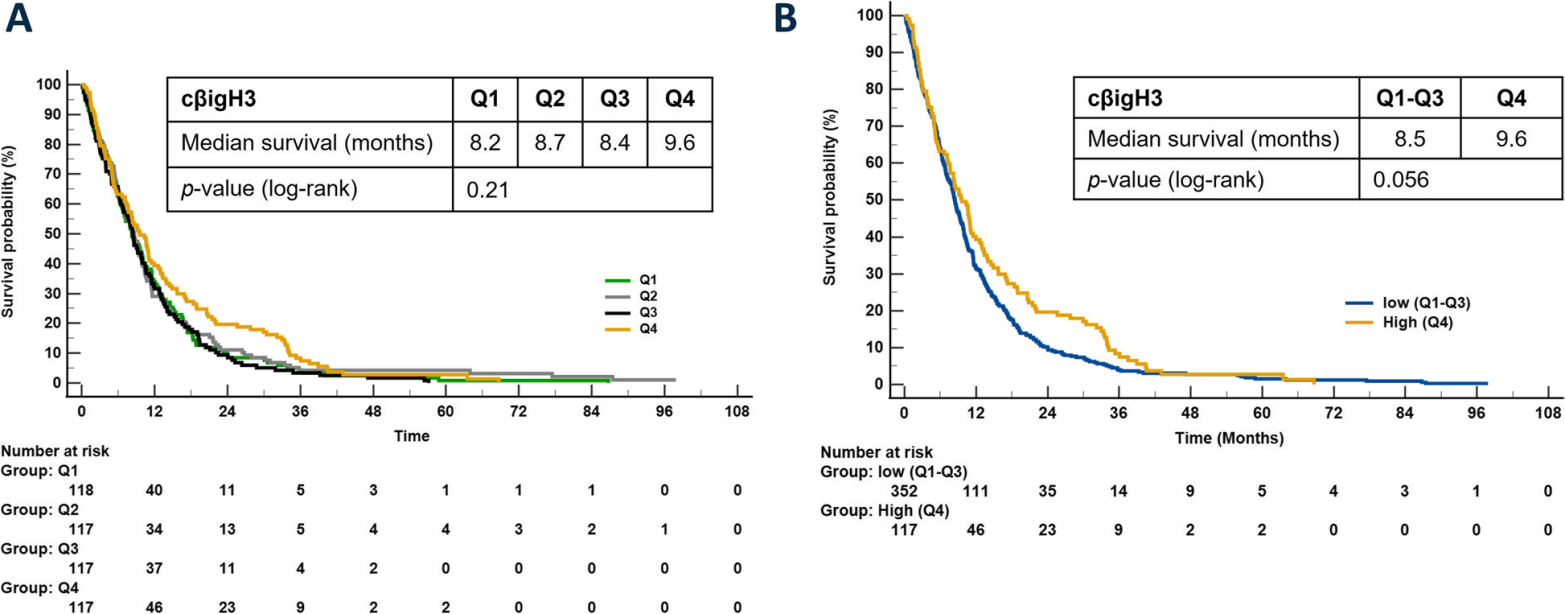
Prognostic biomarker potential of cβigH3 in serum from patients with advanced PDAC. Kaplan–Meier survival curves for overall survival and cβigH3 as a categorical variable, dichotomized in four quartiles (Q1, Q2, Q3, and Q4) (**A**) and in 2 groups separated at the highest quartiles (Q1-3 and Q4) (**B**)

A univariate Cox analysis for cohort 2 showed no significant connection between overall survival outcome and cβigH3 as a continuous variable (HR = 0.94, 95% CI 0.88–1.01, *p* = 0.10) (Table 4). Patients were separated into quartiles based on cβigH3 levels. Median overall survival was 8.2, 8.7, 8.4, and 9.6 months for patients in Q1, Q2, Q3, and Q4, respectively (Fig. 4A). Patients in the three lowest quartiles showed similar survival outcomes, while patients with serum cβigH3 levels in the highest quartile tended to have longer overall survival. We therefore combined patients with serum cβigH3 levels in the lowest three quartiles (Q1-Q3) and compared their survival with the patients in the highest quartile (Q4) and found a trend for high levels of cβigH3 to be associated with longer overall survival (HR = 0.81, 95% CI 0.66–1.006, *p* = 0.06) (Fig. 4B and Table 4).

**Table 4:** Univariate and multivariate Cox proportional-hazard regression analysis for patients with locally advanced or metastatic PDAC (cohort 2)

Univariate Cox proportional-hazard regression analysis			
**Variable**	**Cut point**	**HR (95% Ci)**	***p*-value**
cβigH3	Continuous Q4 vs. Q1-3	0.94 (0.88–1.01) 0.81 (0.66–1.006)	0.10 0.06
PRO-C3	Continuous > median vs. ≤ median	1.001 (1.00–1.001) 1.20 (1.002–1.45)	0.06 0.048
CA19-9	> median vs. ≤ median	1.56 (1.29–1.87)	< 0.0001
Age	Continuous	1.02 (1.01–1.03)	0.001
Sex	Female vs male	1.03 (0.86–1.24)	0.74
BMI	Continuous	0.99 (0.97–1.01)	0.29
Stage	4 vs. 3	1.97 (1.62–2.38)	< 0.0001
Diabetes	Yes vs. no	0.94 (0.76–1.15)	0.55
Tobacco	Never vs Ever	1.00 (0.83–1.22)	0.96
Alcohol	> DHAR vs. < DHAR	1.06 (0.84–1.34)	0.61
PS	1–3 vs. 0	1.69 (1.37–2.08)	< 0.0001
**Multivariate Cox proportional-hazard regression analysis**			
**Variable**	**Cut point**	**HR (95% Ci)**	**p-value**
cβigH3	Q4 vs. Q1-3	0.78 (0.61–0.98)	0.036
Age	Continuous	1.02 (1.00–1.03)	0.016
Stage	4 vs. 3	2.08 (1.68–2.58)	< 0.0001
PS	1–3 vs. 0	1.60 (1.29–1.99)	< 0.0001
CA19-9	> median vs. ≤ median	1.30 (1.06–1.59)	0.011
PRO-C3	> median vs. ≤ median	1.22 (1.00–1.50)	0.053

*Abbreviations: BMI* body mass index, *CA19-9* carbohydrate antigen 19–9, *DHAR* Danish Health Authority recommendations on alcohol consumption, *HR* hazard ratio, *PS* performance status, *Na* not available

PRO-C3 was prognostic in cohort 2, where high levels (> median) of PRO-C3 were significantly associated with short overall survival (HR = 1.20, 95% CI 1.00–1.45, *p* = 0.048) (Table 4). And despite βigH3 and PRO-C3 both being associated with fibrotic phenotypes only a weak correlation between PRO-C3 and cβigH3 levels (r = 0.13, *p* = 0.006) (data not shown). In addition to PRO-C3 (≤ median vs. > median (≤ 89.21 ng/mL vs. > 89.21 ng/mL)), age, performance status (PS) (0 vs. 1–3), CA19-9 levels (≤ median vs. > median (≤ 704 U/mL vs. > 704 U/mL)), was identified as potential confounders related to overall survival that could affect the assessment of prognostic potential of cβigH3 based on univariate Cox-proportional hazard regression (Table 4). We performed a multivariate Cox-proportional hazard regression for high cβigH3 levels (Q4) compared to low cβigH3 levels (Q1-3) adjusting these potential cofounders by multivariate Cox-proportional hazard regression. High cβigH3 levels (Q4) was significantly associated with longer overall survival compared to patients with low levels (HR = 0.78, 95% CI 0.61–0.98, *p* = 0.04) (Table 4) indicating that patients with high cβigH3 levels (Q4) predicted a 22% reduced risk of mortality independent of age, PS, CA19-9, and PRO-C3.

## Discussion

In this study, we developed an ELISA to assess degradation of βigH3 in serum by targeting a peptide fragment generated through proteolytic processing of βigH3 (cβigH3). We demonstrated neoepitope cleavage specificity towards the targeted fragment (cβigH3) and confirmed that it was generated from cleavage of both recombinant βigH3, and fibroblast generated matrices in vitro (Figs. 1 and 2).

Kim et al. [47] identified the cleavage site that generates cβigH3 and found that cleavage of βigH3 influences cell migration while intact βigH3 did not, thus indicating a biological role of the cleavage fragment. With the assay developed in the study we provide a novel tool to further investigate the role of proteolytic cleaved βigH3 that could be used to improve the underlying mechanisms and how it relates to clinical outcome.

Serum cβigH3 had prognostic biomarker potential in patients with locally advanced or metastatic PDAC, where high levels of cβigH3 was significantly associated with longer overall survival compared to patients with low cβigH3 levels independent of age, stage, PS, CA19-9, and PRO-C3. This indicates that degradation of βigH3 is beneficial for patients with advanced PDAC and potentially other fibrotic cancer types. Since βigH3 is a matrix embedded protein and the generation of the cβigH3 fragment is based on ECM remodeling, we investigated the connection between this biomarker and the well-established tumor fibrosis biomarker PRO-C3, where high levels are associated with short overall survival in patients with PDAC [14].

PDAC is often diagnosed at a late stage where treatment options are limited and often unsuccessful. Early stage diagnostic biomarkers for PDAC are key to timely initiation of curative treatment in time [50, 51]. In this study we found no indications of a diagnostic potential for cβigH3 in PDAC or other types of cancer.

Both βigH3 expression and type III collagen formation (reflected by PRO-C3) are induced by TGF-β and are associated with tumor fibrosis, several other fibrotic diseases, and poor patient outcome [14, 52–58]. In this study we found that degradation of βigH3, reflected by high serum cβigH3, was associated with better prognosis independently of PRO-C3. While the exact role of βigH3 in tumor fibrosis is still not fully understood, it could be speculated that its association to poor patient outcome depends on a balance between formation and degradation and could therefore be better assessed by investigating both formation and degradation. For type III collagen proteolytic degradation relative to formation (PRO-C3) shows a stronger associated with better prognosis than the degradation alone [55, 59].

As the use of anti-fibrotic drugs for treatment of cancer are emerging, pharmacodynamic biomarkers for monitoring the effectiveness of the treatment are important [60–62]. With βigH3 expression being related to poor patient outcome across several types of cancer and expected relation between release of cβigH3 and ECM degradation, it could be speculated that increased cβigH3 levels would reflect degradation of tumor-promoting ECM, thus making cβigH3 a potential pharmacodynamic biomarker for anti-fibrotic treatments.

In this study, we asses βigH3 degradation based on a fragment generated from cleavage at a specific cleavage site. ECM remodeling in vivo is more complex and with a large mixture of different proteases with distinct cleavage sites that are related to various biological processes [63–66]. Consequently, despite cβigH3 being directly dependent on processing of βigH3, it may not reflect total degradation of βigH3, as not all degradation can be expected to generate cβigH3.

Furthermore, βigH3 is primarily embedded in the ECM where the matrix would be expected to form a barrier around the protein, preventing degradation from non-collagenase proteases. In contrast, matrix degradation would lead to release of βigH3 proteins from the matrix, leaving them more susceptible to being processed, potentially generating cβigH3 fragments [67]. In that way, cβigH3 could be influenced or potentially dependent on ECM degradation in general.

Our analyses had different limitations for the two cohorts. For cohort 1, the group size for each type of cancer was small and without assessable information of patient outcome, preventing assessment of prognostic biomarker potential. For cohort 2 only patients with locally advanced or metastatic PDAC were included. For both cohorts there were differences in age (mean and range) and/or sex distribution between the patient groups and the healthy subject groups, which could also affect the results as the small sample size might limit the ability to detect small difference that could have biological relevance.

## Conclusion

Proteolytic degradation of βigH3 leads to release of the specific cleaved fragment, cβigH3, that can be quantified in serum with our cβigH3 targeting ELISA. The ELISA was specific towards the targeted neoepitope, and the fragment could be generated from processing of intact βigH3 or decellularize matrix from pancreatic fibroblasts. We showed that cβigH3 could be detected in serum from patients with different types of solid cancer and that high serum levels of cβigH3 were associated with longer overall survival for patients with locally advanced or metastatic PDAC independent of relevant confounders including the tumor fibrosis marker PRO-C3.

## Supplementary Information

Supplementary Material 1

## Data Availability

The data presented in this study can be available upon request and obtained from the corresponding author.

## Abbreviations

βigH3: Beta-Ig H3 / TGF-β induced protein (TGFBI)
CAF: Cancer associated fibroblast
CA19-9: Carbohydrate antigen 19–9
cβigH3: Cleaved βigH3
DHAR: Danish health authorities’ recommendations
ECM: Extracellular matrix
FBS: Fetal bovine serum
HR: Hazard ratio
HRP: Horseradish peroxidase
KLH: Keyhole limpet hemocyanin
LLOQ: Lower limit of quantification
MRD: Minimum required dilution
PDAC: Pancreatic ductal adenocarcinoma
PS: Performance status
P/S: Penicillin/streptavidin
RE%: Relative error percent
rpm: Revolutions per minute
TGF-β: Transforming growth factor beta
TME: The tumor microenvironment
ULOQ: Upper limit of quantification
