# Supplementary material for: Proteolytic degradation of Beta-Ig H3 (βigH3/TGFBI) can be quantified non-invasively in serum and predicts prognosis in patients with advanced pancreatic ductal adenocarcinoma
Source: BMC Cancer. 2025 May 20;25:905. doi: 10.1186/s12885-025-14283-w (PMC12093888; doi:10.1186/s12885-025-14283-w)
Supplement: Supplementary file 1 — Supplementary Material 1 [file 12885_2025_14283_MOESM1_ESM.docx]

**Supplementary figure 1 LLOQ determination.** Four serum samples with low levels of cβigH3 were measured in five independent runs with six replicates in each mean values and CV% are plotted. A power regression was made based on these values ($y=C_{0}x^{c_{1}}$) and from this function LLOQ was determined as the concentration with 20% CV ($x={\frac{y}{C_{0}}}^{1/C_{1}}$).
